# C/EBPδ Suppresses Motility-Associated Gene Signatures and Reduces PDAC Cell Migration

**DOI:** 10.3390/cells11213334

**Published:** 2022-10-22

**Authors:** Leonie Hartl, Pien A. F. Maarschalkerweerd, Joe M. Butler, Xue D. Manz, Victor L. J. L. Thijssen, Maarten F. Bijlsma, JanWillem Duitman, C. Arnold Spek

**Affiliations:** 1 Laboratory for Experimental Oncology and Radiobiology, Center for Experimental and Molecular Medicine, Amsterdam UMC Location University of Amsterdam, 1105 AZ Amsterdam, The Netherlands; 2 Cancer Center Amsterdam, Cancer Biology and Immunology, 1081 HV Amsterdam, The Netherlands; 3 Department of Pulmonary Medicine, Amsterdam UMC Location VU University Medical Center, 1081 HV Amsterdam, The Netherlands; 4 Department of Radiation Oncology, Amsterdam UMC Location VU University Medical Center, 1081 HV Amsterdam, The Netherlands; 5 Department of Pulmonary Medicine, Amsterdam UMC Location University of Amsterdam, 1105 AZ Amsterdam, The Netherlands; 6 Department of Experimental Immunology, Amsterdam UMC Location University of Amsterdam, 1105 AZ Amsterdam, The Netherlands; 7 Amsterdam Infection & Immunity, Inflammatory Diseases, 1105 AZ Amsterdam, The Netherlands

**Keywords:** CCAAT/enhancer-binding protein delta, pancreatic ductal adenocarcinoma, metastases, migration, cytoskeleton

## Abstract

Pancreatic Ductal Adenocarcinoma (PDAC) is among the most aggressive human cancers and occurs globally at an increasing incidence. Metastases are the primary cause of cancer-related death and, in the majority of cases, PDAC is accompanied by metastatic disease at the time of diagnosis, making it a particularly lethal cancer. Regrettably, to date, no curative treatment has been developed for patients with metastatic disease, resulting in a 5-year survival rate of only 11%. We previously found that the protein expression of the transcription factor CCAAT/Enhancer-Binding Protein Delta (C/EBPδ) negatively correlates with lymph node involvement in PDAC patients. To better comprehend the etiology of metastatic PDAC, we explored the role of C/EBPδ at different steps of the metastatic cascade, using established in vitro models. We found that C/EBPδ has a major impact on cell motility, an important prerequisite for tumor cells to leave the primary tumor and to reach distant sites. Our data suggest that C/EBPδ induces downstream pathways that modulate actin cytoskeleton dynamics to reduce cell migration and to induce a more epithelial-like cellular phenotype. Understanding the mechanisms dictating epithelial and mesenchymal features holds great promise for improving the treatment of PDAC.

## 1. Introduction

When combining all tumor stages at the time of diagnosis, Pancreatic Ductal Adenocarcinoma (PDAC) has the lowest 5-year survival rate of all cancers, which is now 11% [1]. Currently, PDAC is the third-leading cause of cancer-related mortality in the United States (US) and predicted to become the second-leading cause of cancer-related death by 2040 [1,2]. Although not a common cancer, PDAC incidence is increasing between 0.5 and 1% every year, which resulted in around 60,430 new cases in 2021 in the US alone. A mere 10–20% of patients present with resectable disease, whilst 80–90% are diagnosed with either locally advanced or metastatic disease, leading to a median survival of 6–11 and 3–6 months, respectively [3,4,5]. Metastatic disease and local and metastatic relapse after resection are considered the main cause of mortality in cancer. PDAC is especially prone to yield metastases, urging a better understanding of the mechanisms underlying metastatic development in this disease [6].

For primary tumor cells to establish metastatic lesions, a series of steps—referred to as the metastatic cascade—must be completed [7]. To initiate the metastatic cascade, the epithelial-like cancer cells at the invasive front of the tumor undergo morphological and, presumably, genetic changes, shifting them into a mesenchymal-like state [8]. Through this transition, cells lose cell–cell junctions and trade their epithelial-like shape for a spindle-like shape accompanied by invadopodia, allowing them to disseminate from the primary tumor. Simultaneously, an upregulation of matrix-degrading enzymes allows them to invade the surrounding stroma and to eventually reach nearby vessels [9]. Intravasation of lymph or blood vessels constitutes the next crucial step of the metastatic cascade, which is accompanied by further genetic and epigenetic changes as well as metabolic adaptions and is often aided by different cell types of the tumor microenvironment [10,11]. Once cells have reached the blood stream, circulating tumor cells need to escape from anoikis and to survive high shear stresses as well as immune surveillance [12]. Through the activation of tumor-specific ligands and receptors, circulating tumor cells can then attach to and modulate endothelial cells in order to extravasate through the endothelial layer and the surrounding basement membrane at distant locations to colonize metastatic niches [12,13]. Whether in PDAC, tumor cells colonize distant tissues before a clinically detectable primary tumor mass is established, is still under debate [6].

CCAAT/Enhancer-Binding Protein Delta (C/EBPδ) is a transcription factor of the C/EBP-family that currently comprises C/EBPα, -β, -γ, -δ, -ε, and -ζ. The different family members form homo- or heterodimers that bind specific DNA sequences and induce the transcription of a plethora of genes associated with differentiation processes, cell cycle regulation, proliferation, and apoptosis. Next to this, C/EBPδ is currently under close scrutiny in different cancers and an image emerges of a highly context-dependent role of C/EBPδ in carcinogenesis and metastasis formation. The knockdown of *CEBPD* in urothelial carcinoma cells limited cell proliferation, migration, and invasion [14]. On the contrary, we recently described an inverse correlation between C/EBPδ expression in primary tumor cells and their ability to form lymph node metastases in PDAC [15]. Briefly, lower nuclear C/EBPδ tumor cell expression associated with an increased likelihood of lymph node metastases and a decreased median overall survival. Concurrently, C/EBPδ is lower expressed in PDAC cells as opposed to normal pancreatic ductal cells [15].

To investigate the relationship between C/EBPδ and metastasis formation in PDAC, we established PDAC cell lines with a doxycycline-inducible C/EBPδ expression. We previously found that the induction of C/EBPδ in these cells limits their proliferative capacity, implying a tumor-suppressor role in PDAC. As decreased tumor cell proliferation does not necessarily account for decreased lymph node involvement, we herein used this system to subject PDAC cells to various in vitro assays modeling different steps of the metastatic cascade.

## 2. Materials and Methods

### 2.1. Cell Lines and Cell Culture Reagents

MIA PaCa-2 (CRL-1420, ATCC, Manassas, VA, USA) and PANC-1 (CRL-1469, ATCC) cells were maintained in DMEM medium (#41965120, Gibco, Waltham, MA, USA) supplemented with 10% (*v*/*v*) fetal calf serum (#S-FBS-NL_015, Serana, Pessin, Germany), 2% (*v*/*v*) penicillin–streptomycin (#15140122, Gibco), and 2 mM L-glutamine (#17-605E, Lonza, Basel, Switzerland), hereafter referred to as complete growth medium, at subconfluency in a tissue culture incubator in 5% CO_2_ at 37 °C. Cells were monthly tested negative for mycoplasma and their identity was confirmed yearly by STR profiling (last in February 2022).

### 2.2. Cloning Strategy and Lentiviral Transduction

Control (CTRL) and C/EBPδ-inducible cells were prepared as described previously [15]. Briefly, MIA PaCa-2 (CRL-1420, ATCC) and PANC-1 cells (CRL-1469, ATCC) were transduced with the *pCW57* vector (#80921, Addgene, Watertown, MA, USA) bearing either a doxycycline-inducible transactivator (tTA) and TRE promoter and the *CEBPD* cDNA (C/EBPδ-inducible cells) or the tTA and TRE promoter without *CEBPD* cDNA (CTRL cells). Additionally, to distinguish C/EBPδ-inducible cells from CTRL cells, green and red fluorophores were added through lentiviral transduction with LeGO-V2 (*mVenus*, #27340, Addgene) and LeGO-C2 (*mCherry*, #27339, Addgene), respectively [16]. A pool of three C/EBPδ-inducible clones was used for downstream experiments.

### 2.3. RNA Extraction and RNA Sequencing

Cells (3.5 × 10^5^) per well were seeded in 12-well plates in triplo and either treated with doxycycline at a concentration of 2 µg/mL to induce C/EBPδ expression for 24 h or without doxycycline to study effects of baseline C/EBPδ expression. Cells were then lysed and RNA was extracted using the NucleoSpin RNA-extraction kit (#740955, Macherey-Nagel, Düren, Germany) according to the supplier’s protocol for cultured cells. RNA quality was assessed by bioanalysis (TapeStation, Agilent), with all samples having RNA integrity numbers (RINs) > 7. Total RNA concentrations were determined by Qubit 2.0 Fluorometer (Life Technologies, Waltham, MA, USA). Sequencing libraries were prepared by means of the KAPA RNA HyperPrep with RiboErase (#KR1351—v1.16, Roche, Woerden, The Netherlands) as per manufacturer’s instructions. Libraries were sequenced using the Illumina HiSeq4000 (Illumina) to generate 50 bp reads.

### 2.4. Bioinformatic Analyses

RNA sequence read quality was assessed using FastQC methods (version 0.11.5; Babraham Institute, Cambridge, UK). Trimmomatic version 0.39 [17] was used to trim the Illumina adapters and to filter low-quality reads and ambiguous nucleotide-containing sequences. Low-quality nucleotides (3 leading and 3 trailing) were removed from each read. A sliding window trimming using a window of 4 and a Phred score threshold of 15 nucleotides was used to access the quality of the reads. After pre-processing, the remaining high-quality reads were aligned against the human Genome Reference Consortium Build 38 (GRCh38, Ensembl 84) using Hisat2 (2.2.0) [18] with default parameters. Count data were generated by means of the HTSeq method [19]. The R2 Genomics Analysis and Visualization Platform [20] was used to determine differentially expressed genes between samples with baseline *CEBPD* expression and samples in which *CEBPD* was induced for 24 h using ANOVA with a cutoff at FDR-corrected *p* < 0.01. Volcano plots were generated using the R2 Genomics Analysis and Visualization Platform [20] and annotated using GraphPad Prism (version 9.1.0, GraphPad Software Inc., San Diego, CA, USA). Gene Ontology (GO) term enrichment analysis was done using the GOrilla Gene Ontology Enrichment Analysis and Visualization Tool [21,22], applying the Biological Process-Ontology. Gene Set Enrichment Analysis (GSEA) was performed using the Broad Institute GSEA tool (version 4.2.1) [23,24]. The median CEBPD expression of all samples was used as the threshold to dichotomize the samples. Signal-to-noise was selected as the metric for ranking genes. *p*-values indicating the significance of enrichment were determined by 1000 permutations using a gene set comprising actin dynamics-associated gene sets retrieved from the MSigDB Broad Institute gene set database: GOBP: positive regulation of actin filament bundle assembly (GO:0032233), GOBP: positive regulation of actin filament depolymerization (GO:0030836), GOBP: positive regulation of actin cytoskeleton reorganization (GO:2000251), GOBP: positive regulation of actin nucleation (GO:0051127), and GOBP: positive regulation of actin filament polymerization (GO:0030838). Duplicate genes were removed such that each gene is represented only once in the final gene set.

### 2.5. Scratch Migration Assays

PDAC cells transduced with doxycycline-inducible *pCW-CEBPD* (and *mVenus* fluorophore) or *pCW-CTRL* (and *mCherry* fluorophore) constructs were mixed at equal ratios and seeded at 20,000 cells per well in the Incucyte Imagelock 96-well plate (#BA-04856, Sartorius, Göttingen, Germany) and allowed to attach overnight in complete medium. Doxycycline was added to half of the wells at a concentration of 2 µg/mL. The next day, cell layers were wounded using the Incucyte wound maker tool (#4563 and #5025-0191, Sartorius), the cells were washed once with PBS, and fresh complete medium with 1.5–2.5% FCS and with or without doxycycline was added and refreshed every 2–3 days. Cells were imaged at regular intervals using the Incucyte S3 Live-Cell Analysis Instrument (Sartorius). The Incucyte Scratch Wound Analysis Software Module (#9600-0012, Sartorius) was used to quantify the percentage of initial wound area covered by red fluorescent or green fluorescent cells. To correct for differences in baseline migration between CTRL and C/EBPδ-inducible cells, the following formula was used: C/EBPδ_doxycycline(corrected)_ = C/EBPδ_doxycycline_ ∗ (CTRL_untreated_/C/EBPδ_untreated_). Graphs and linear regression to compare migration slopes were done using GraphPad Prism (version 9.1.0, GraphPad Software Inc.).

### 2.6. Manual Cell Tracking

Cells were seeded at 100 cells per well in 24-well plates and imaged once per hour using the Incucyte S3 Live-Cell Analysis Instrument (Sartorius). The exported time lapse images were imported into ImageJ [25] and the Manual Tracker-Plugin was used to follow the most motile cells in each condition by marking the cell’s center at each consecutive time point. The results table was exported and graphs and Mann–Whitney U tests were performed using GraphPad Prism (version 9.1.0, GraphPad Software Inc.).

### 2.7. Chemotaxis Assays

For chemotaxis assays, 2 × 10^4^ PDAC cells were seeded in the Incucyte Clearview 96-well Plate for chemotaxis (#4582, Sartorius) in medium supplemented with 1% FCS. Half of the wells were treated with doxycycline 2 μg/mL, which was refreshed every 2–3 days. Bottom compartments of the wells contained complete growth medium. The Incucyte S3 Live-Cell Analysis Instrument (Sartorius) was used to image cells at 4-h intervals and the Incucyte Chemotaxis Analysis Software Module was used to quantify the cell-covered area at the bottom of the transwell membrane over time. GraphPad Prism (version 9.1.0, GraphPad Software Inc.) was used to reproduce migration curves.

### 2.8. Fluorescence-Activated Cell Sorting (FACS)

PDAC CTRL and C/EBPδ-inducible cells were treated or not with doxycycline 2 µg/mL for up to 4 weeks and regularly trypsinized and resuspended in FACS buffer (1% FCS in PBS) for FACS analysis (CytoFLEX S Flow Cytometer, Beckman Coulter, Brea, CA, USA). Cell populations were analyzed using the CytExpert Acquisition and Analysis Software Version 2.4. Instrument settings were calibrated before each use using the CytoFLEX Daily CQ Fluorospheres (#B53230, Beckman Coulter). 

### 2.9. Phalloidin Staining

MIA PaCa-2 cells were seeded at 10,000 cells per well in 8-well chambered coverslides (Nunc Lab-Tek, #155383, Waltham, MA, USA), treated or not with doxycycline at 2 µg/mL, and allowed to attach for 48 h. Cells were washed with PBS, fixed in 4% paraformaldehyde (ProSciTech, #C006) and permeabilized with 0.1% Triton X-100 (MERCK, X-100, Darmstadt, Germany) for 10 min at room temperature each and stained using ActinGreen 488 Ready Probes Reagent (Invitrogen, #R37110, Waltham, MA, USA) according to the manufacturer’s protocol. Cells were imaged using the Leica Digital LightSheet confocal microscope TCS SP8 DLS.

### 2.10. Gene Knockdown

Per target gene, five short hairpin RNAs (shRNA) (MISSION shRNA library, MERCK) were used for lentiviral silencing. Based on RT-qPCR analysis, cells transduced with the following shRNA clones were used for downstream experiments: Neuroepithelial Cell Transforming 1 (*NET1*, NM_005863, TRCN0000047763), Gelsolin (*GSN*, NM_000177, TRCN0000029724), Myopalladin (*MYPN*, NM_032578, TRCN0000073454), Integrin Subunit Beta 4 (*ITGB4*, NM_000213, TRCN0000057771), AXL Receptor Tyrosine Kinase (*AXL*, NM_001699, TRCN0000001037), Epithelial Growth Factor Receptor (*EGFR*, NM_005228, TRCN0000121069), and ETS Proto-Oncogene 1 (*ETS1*, NM_005238, TRCN0000005588) (Figure S1). For RT-qPCR, knockdown cells were lysed and RNA was extracted using the NucleoSpin RNA-extraction kit (#740955, Macherey-Nagel) according to the supplier’s protocol for cultured cells. The eluted RNA was analyzed spectrophotometrically using the NanoDrop 2000 (#ND-2000, Thermo Scientific, Waltham, MA, USA). All samples were treated with RQ1 RNAse-Free DNAse (#M6101, Promega, Madison, WI, USA) and reverse-transcribed into cDNA using M-MLV Reverse Transcriptase (#M1701, Promega), random hexamers (#N8080127, Fisher Scientific), and 10 mM dNTPs (#R0192, Fisher Scientific). The SensiFAST SYBR No-ROX Kit (#BIO-980, GC biotech) was used to perform real-time quantitative RT-qPCR in a LightCycler 480 Instrument (Roche). Gene expression levels were normalized to the expression of the reference genes *TBP* and *RPLP0* using the primers enlisted in Table S1.

## 3. Results

### 3.1. C/EBPδ Induces Migration-Associated Gene Signatures

**Figure 1:**
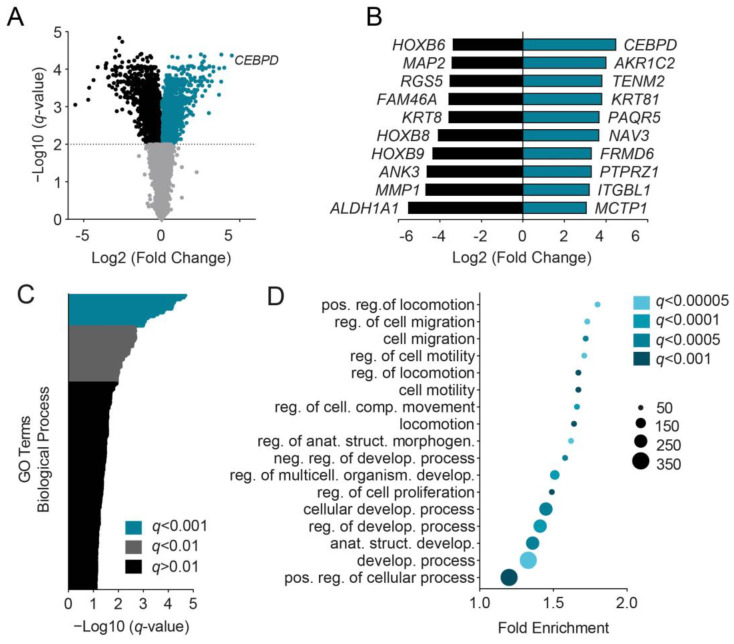
Genes induced by CCAAT/Enhancer-Binding Protein Delta (C/EBPδ) correlate with migration-associated Gene Ontology (GO) terms. (**A**) A total of 24 h after C/EBPδ induction, differentially expressed genes (DEGs, N = 3768) were determined using ANOVA. The volcano plot shows the fraction of significantly upregulated (turquoise) and downregulated (black) genes and genes that were not significantly affected by C/EBPδ (gray) (FDR-corrected *p* < 0.01). Plotted is the Log_2_ of the fold change over the gene expression in the absence of C/EBPδ. (**B**) The 10 most enriched (turquoise) and 10 most depleted (black) genes were extracted from all 3768 DEGs. (**C**) Differentially expressed genes (same DEGs as in panel (**A**)) were extracted to find enriched GO terms in the domain Biological Process. A total of 157 GO terms were found to be significantly enriched (turquoise and gray) and are ordered on the *y*-axis. The *x*-axis shows the negative Log_10_ of the *p*-value corrected for multiple testing (FDR *q*-value). (**D**) GO terms with *q* < 0.001 were extracted from Figure 1C to visualize the fold enrichment, reporting the significance of the enrichment (*q*-value) and the number of genes in the intersection of DEGs and each GO term. Abbreviations: FDR—false discovery rate.

We previously showed that a high expression of C/EBPδ in primary tumor cells correlates with a decreased likelihood of lymph node involvement in PDAC patients [15]. In the current study, we aimed to delineate downstream pathways regulated by C/EBPδ that potentially contribute to this phenomenon. To this end, C/EBPδ expression was induced in MIA PaCa-2 cells using a doxycycline-responsive Tet-On system [15]. Subsequently, RNA-sequencing was performed on ribosomal RNA (rRNA)-depleted total RNA after 24 h of doxycycline treatment. To account for changes in gene expression caused by doxycycline, non-inducible cells simultaneously treated with doxycycline were used as the reference group. Using ANOVA and applying a cut-off of an FDR-corrected *p* < 0.01, a total of 3768 differentially expressed genes (DEGs) between control (CTRL) cells and C/EBPδ-high cells were found (Figure 1A,B). A total of 1564 of those DEGs were overexpressed upon the induction of C/EBPδ, and 2204 were suppressed. The resulting gene list was ranked by *q*-value (FDR-corrected *p*-value) and subjected to Gene Ontology (GO) term enrichment analysis using GOrilla [21]. This analysis revealed 157 significantly (*q* < 0.01) enriched GO terms (Figure 1C and File S1). From these, GO terms with a highly significant enrichment of *q* < 0.001 were extracted and their fold enrichment, together with the number of genes in the intersection of DEGs and the genes in the GO term, was determined (Figure 1D).

From the GO term enrichment analysis, it was apparent that the expression of C/EBPδ specifically induced transcriptional changes associated with cell motility (locomotion, cell migration, motility, and movement) as well as terms associated with cellular and organismal development. Cell motility is indeed an important factor in the ability of tumor cells to disseminate from the primary tumor and to initiate the formation of distant metastases. Thus, we next investigated the effect of C/EBPδ on PDAC cell migration.

### 3.2. Induction of C/EBPδ Expression Reduces Migration in PDAC Cells

**Figure 2:**
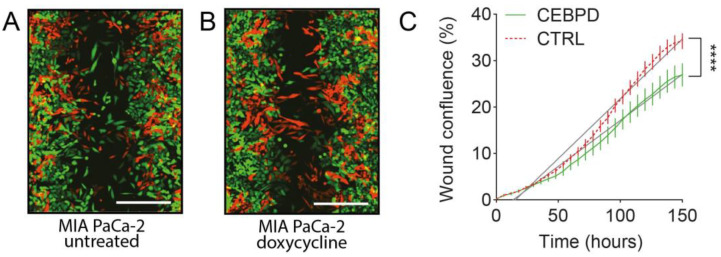
Scratch-wound assays reveal that C/EBPδ slows migration in MIA PaCa-2 cells. MIA PaCa-2 cells inducible for C/EBPδ expression through addition of doxycycline (green fluorescent) and non-inducible control (CTRL, red fluorescent) cells are mixed at equal ratios and subjected to a scratch-wound assay. (**A**) Without doxycycline treatment, C/EBPδ-inducible and CTRL cells migrate into the wound area at similar rates. (**B**) The induction of C/EBPδ expression by doxycycline (green fluorescent cells) ceases migration, while CTRL cells (red fluorescent cells) migrate efficiently into the wound area. Scale bars are 400 µm. (**C**) Cells were imaged at six-hour time intervals and an image analysis software was used to quantify the percentage of wound area covered by each respective cell type. Shown is a representative migration profile of CTRL (red fluorescent, red dotted line) and C/EBPδ-inducible (green fluorescent, green full line) cells in the presence of doxycycline. Data were normalized to untreated cells. Vertical bars represent the standard error of the mean of six replicates. Straight lines were fit by simple linear regression that showed that the slopes of both curves are significantly different (**** *p* < 0.0001). The experiment was repeated four times with at least six replicates.

Wound-healing assays are a well-established tool to quantify the motility of cancer cells. To test the effect of C/EBPδ expression on the migratory capacity of PDAC cells, C/EBPδ was induced in MIA PaCa-2 and PANC-1 cells through the doxycycline-inducible construct described above. Inducible and CTRL cells were combined at equal ratios and either treated with doxycycline or left untreated. After wounding, the cells were followed for five days, by which time the cells had closed the wound in all conditions. Figure 2 shows representative images of the wound area three days after wounding in the absence (Figure 2A) and presence (Figure 2B) of doxycycline. In the untreated condition, red CTRL cells and green C/EBPδ-inducible cells migrate into the scratch at similar rates. However, when C/EBPδ is induced through doxycycline in the green cells, they cease to migrate and remain at the edge of the wound. Figure 2C shows representative migration curves of CTRL and C/EBPδ-inducible cells in the presence of doxycycline. Indeed, when C/EBPδ is induced, migration is significantly reduced in MIA PaCa-2 cells (*p* < 0.0001). Of note, in less motile PANC-1 cells, the induction of C/EBPδ had no effect on migration (Figure S2).

**Figure 3:**
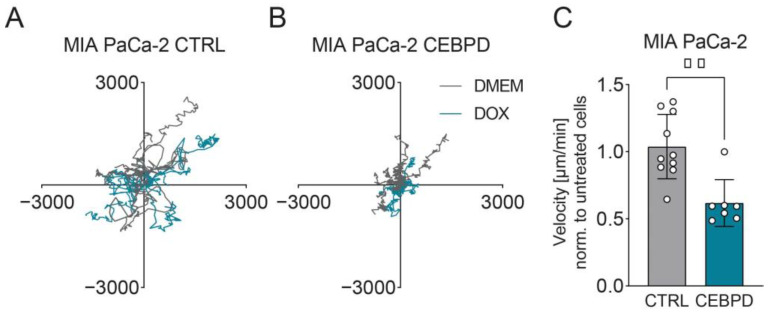
C/EBPδ reduces random single-cell migration in MIA PaCa-2 cells. (**A**,**B**) Single cell migration tracks of multiple CTRL (**A**) or C/EBPδ-inducible (**B**) cells with (turquoise lines) or without (gray lines) doxycycline-treatment migrating freely on plastic for three days, plotted in µm (*x*- and *y*-axis). For tracking, the fastest moving cells in each condition were selected. (**C**) Shown is the velocity of CTRL and C/EBPδ-inducible cells in the presence of doxycycline normalized to the velocity of the respective untreated cells. The decline in migration caused by C/EBPδ induction is significant at ** *p* < 0.01 (Mann–Whitney U test).

A drawback of scratch-wound assays is that they cannot entirely rule out the effects of proliferation on wound closure. Thus, we next tested the effects of C/EBPδ on random single-cell migration. To this end, cells were sparsely seeded and imaged at one-hour intervals for three days to cover multiple cell-division cycles. Spider graphs in Figure 3A,B show the random migration tracks of multiple CTRL cells and doxycycline-inducible cells in the presence or absence of doxycycline in the 2-dimensional environment. Due to an intrinsic heterogeneity, cells from a seemingly homogeneous population often move at different speeds, introducing cellular and temporal noise [26]. As faster moving cells are considered more carcinogenic, we focused our analysis on the most motile cells to see whether C/EBPδ can indeed reduce their migration velocity. Figure 3C shows the resulting velocity achieved by CTRL and C/EBPδ-inducible cells in the presence of doxycycline. While CTRL cells migrate at a velocity of 0.76 and 0.78 µm per minute in the absence or presence of doxycycline, respectively, C/EBPδ-inducible cells showed a slower baseline migration speed of 0.53 µm per minute which we attribute to an enhanced baseline expression of *CEBPD* mRNA in these cells, probably resulting from the leakiness of the Tet-On-construct (Figure S3) [27]. Upon the induction of C/EBPδ, migration in these cells is further reduced to 0.33 µm per minute, which constitutes a significant decline in migration velocity, supporting the notion that C/EBPδ limits the migratory ability of MIA PaCa-2 cells.

**Figure 4:**
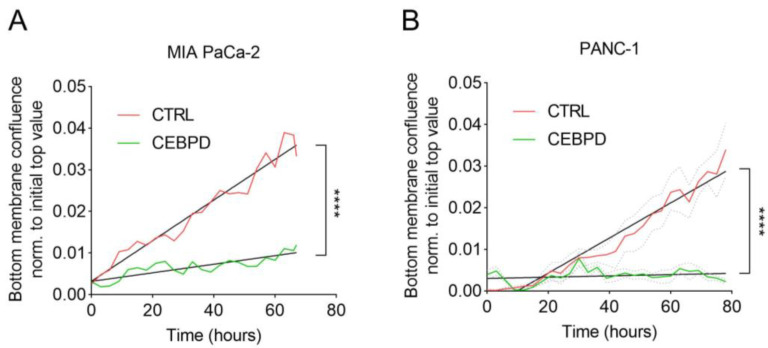
C/EBPδ limits FCS-induced chemotactic transwell migration in MIA PaCa-2 and PANC-1 cells. Induction of C/EBPδ significantly reduced migration along an FCS-gradient in C/EBPδ-inducible MIA PaCa-2 (**A**) and PANC-1 cells (**B**) (green lines) compared with CTRL cells in the presence of doxycycline (red lines). Data were normalized to untreated cells. Straight lines were fit by simple linear regression and show that the slopes of both curves are significantly different in both cell lines (**** *p* < 0.0001). Dotted lines show the standard error of the mean.

Again, random migration of PANC-1 cells was unaffected by C/EBPδ in the single-cell migration assay (Figure S4). From our own observations, we have noticed that individual PANC-1 cells are less likely to move away from their own colony and that their migration pattern rather constitutes a mingling motion with fellow cells of the same colony. To better assess the effects of C/EBPδ on the migration of PANC-1 cells, we next subjected them to a chemotaxis assay using a chemotactic gradient of FCS to stimulate cell migration through a porous transwell membrane. As expected, the induction of C/EBPδ in MIA PaCa-2 cells restrains their ability to migrate through the pores of the transwell membrane and to reach the bottom side of the well (Figure 4A). Additionally, in PANC-1 cells, C/EBPδ had a significant limiting effect on chemotactic migration (Figure 4B).

Altogether, these results show that C/EBPδ reduces the motility of PDAC cell lines, which might ultimately account for the inverse correlation of C/EBPδ protein expression in primary tumors with lymph node involvement in PDAC patients.

To investigate whether C/EBPδ also plays a role at other steps of the metastatic cascade, i.e., invasion or extravasation, we performed in vitro transwell invasion assays using MIA PaCa-2 and PANC-1 cells in Matrigel-coated transwells and subjected the cells to an FCS gradient. No significant differences were observed between the invasiveness of CTRL cells and C/EBPδ-expressing cells (Figure S5). These data were confirmed with *in vivo* chicken chorioallantoic membrane (CAM) assays, where MIA PaCa-2 cells were seeded on the CAM in Matrigel and invasion was followed microscopically by cryosectioning the CAM at three consecutive days (Figure S6). In this model, MIA PaCa-2 cells invaded rapidly through the upper layer of the CAM towards the underlying blood vessels, but again we could not confirm a difference in invasion speed between CTRL and C/EBPδ-high cells. No tumor cells were found in the embryo’s liver or lungs by qPCR or FACS analysis for mCherry (CTRL cells) and mVenus (inducible cells), respectively (data not shown).

We next determined the effect of C/EBPδ on tumor cell extravasation using µ-slide flow chambers. Channels in the flow chambers were coated with primary human Pulmonary Microvascular Endothelial Cells (PMVEC), after which MIA PaCa-2 cells (CTRL and inducible, pre-treated with doxycycline for C/EBPδ induction or not) were run through the channels (Figure S7A,B) [28]. Fluorescence live imaging revealed that MIA PaCa-2 rarely attach to the endothelium and that the induction of C/EBPδ did not affect this behavior in either direction. Additionally, cells failed to extravasate through the endothelial monolayer, irrespective of the induction of C/EBPδ. Additional ECIS experiments showed that the induction of C/EBPδ did not lead to enhanced endothelial monolayer disruption (Figure S7C) [29]. We thus assume that the relation between tumor cell C/EBPδ and lymph node metastases is mostly determined by the effect of C/EBPδ on cell motility. Therefore, we more closely examined how C/EBPδ affects cell migration and motility in PDAC.

### 3.3. C/EBPδ Induces a Gene Signature That Negatively Correlates with Actin Dynamics

Alterations in cancer cell motility are often accompanied by a transition of cells from an epithelial towards a mesenchymal state or vice versa (EMT/MET). To assess whether C/EBPδ induces changes in the expression of epithelial or mesenchymal markers, we tested the expression of an EMT panel in MIA PaCa-2 cells before and after the induction of C/EBPδ (Figure S8). From these data, it became apparent that EMT markers are not coherently changed and, consequentially, C/EBPδ does not reduce migration in PDAC cells through the suppression of an EMT signature.

**Figure 5:**
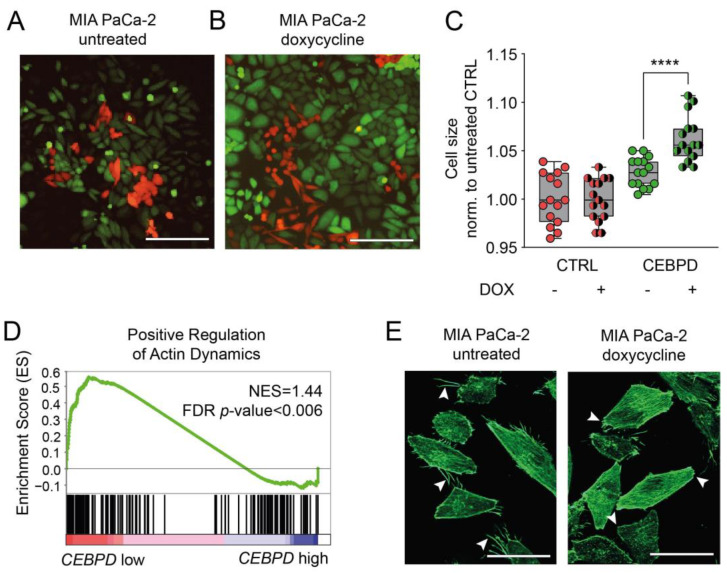
C/EBPδ includes genes associated with the actin cytoskeleton and alters cell morphology. (**A**) C/EBPδ-inducible (green fluorescent) and CTRL (red fluorescent) MIA PaCa-2 cells in cell-growth medium without doxycycline. Cells containing a C/EBPδ-induction construct show slight morphological alterations, i.e., begin to lose their classical spindle shape. Scale bars are 200 µm. (**B**) Upon the addition of doxycycline and the induction of C/EBPδ, this morphology is more enhanced and resembles a cobblestone-like morphology in the green fluorescent cells. (**C**) Data obtained from fluorescence activated cell sorting. Plotted is the forward scatter area which indicates differences in cell size in the different cell lines and conditions. Induction of C/EBPδ leads to significantly enlarged cells (**** *p* < 0.0001, Mann–Whitney U test). (**D**) Gene set expression analysis reveals a significant negative correlation of gene associated with the positive regulation of actin dynamics with *CEBPD*-associated genes. (**E**) Phalloidin staining of MIA PaCa-2 cells before (**left**) and after the induction of C/EBPδ (**right**). White arrowheads point out filopodia extending from different cells after doxycycline treatment or the lack of those in untreated cells. Scale bars are 35 µm.

Irrespective of the apparent lack of an epithelial transition, we observed that C/EBPδ-high cells acquire an altered morphology when C/EBPδ is induced. Wild-type MIA PaCa-2 cells comprise two morphologies, namely spindle-shaped cells and round cells. From time-lapse videos, we learned that spindle-shaped cells become round and detach prior to cell division (red cells in Figure 5A). As described above, cells transduced with a Tet-On construct for C/EBPδ expression show some leakiness of this construct even in the absence of doxycycline (Figure S3) and partly lose their classical spindle shape and the ability to detach properly (green cells in Figure 5A). When C/EBPδ is induced, this effect was more pronounced; cells appeared larger in size and took on a cobblestone-like morphology (green cells in Figure 5B). Non-inducible cells did not show these properties in the presence of doxycycline (red cells in Figure 5B). Adding to these morphological changes, FACS analysis was performed, which revealed that cell size increased with *CEBPD* mRNA expression. Cell morphology and cell size are directly determined by the activity of the cytoskeleton. Interestingly, it has been shown previously that the knockdown of *CEBPD* lead to smaller cells and a more star-shaped morphology in mouse embryonal fibroblasts [30]. Although the underlying mechanisms are not yet clear, these findings implicate that C/EBPδ alters the actin cytoskeleton dynamics in the setting of fibroblasts, which may also apply to PDAC cells. This would mean that with increasing C/EBPδ expression, the cytoskeleton loses its ability to contract and thereby limits migration (Figure 5C). We confirmed this observation by running a Gene Set Enrichment Analysis (GSEA) using our RNA-seq data and a combination of publicly available actin dynamics-related datasets (Table S2). We indeed found a significant negative correlation of gene sets associated with the positive regulation of actin and genes associated with high *CEBPD* expression (*p* = 0.0058) (Figure 5D). Next to this, we observed a moderate decline in the number and the length of filopodia, as well as the number of cells expressing filopodia upon activation of C/EBPδ (Figure 5E). Together, these findings suggest that C/EBPδ alters the expression of downstream targets that modulate the cytoskeleton.

### 3.4. C/EBPδ Suppresses EGFR and GSN to Reduce MIA PaCa-2 Cell Migration

**Figure 6:**
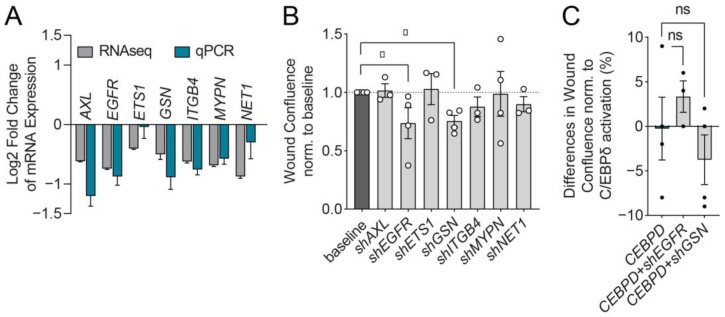
Knockdown of migration-associated targets genes of C/EBPδ. (**A**) Different migration-associated genes that were significantly differentially regulated upon the induction of C/EBPδ were manually selected from RNAseq data. RT-qPCR was used to validate the deregulation of these genes. Shown is the Log_2_ fold change of gene expression over untreated cells. (**B**) Cell lines containing shRNAs against individual genes were established from the C/EBPδ-inducible MIA PaCa-2 cell line and subjected to migration assays (N = 6, 3–4 assays per cell line). C/EBPδ was not induced in these cell lines and the effect of the respective gene knockdown on migration, normalized to the parental cell line (baseline), is shown. Knockdown of Epidermal Growth Factor Receptor (*EGFR*) and Gelsolin (*GSN*) induces a significant reduction of cell migration (* *p* < 0.05). (**C**) C/EBPδ was induced in *shEGFR* and *shGSN* cells to observe whether shRNA-mediated knockdown in these cells causes an additional migration-inhibiting effect together with C/EBPδ-mediated suppression of *EGFR* and *GSN*. No significant additional effect of gene knockdown was observed. ns—not significant.

To investigate potential downstream targets of C/EBPδ associated with the above delineated cellular properties, we next selected a small array of genes that were found most frequently in significantly enriched GO terms and gene sets associated with the actin cytoskeleton, including locomotion, contraction, and cell shape (data shown in Figure 1D and Figure 5D). Genes were further selected based on a proven association with migration in the current literature [31,32,33,34,35,36,37,38,39]. The expression of these genes was confirmed by Real-Time Polymerase Chain Reaction (RT-qPCR) in MIA PaCa-2 (Figure 6A). Interestingly, all these genes were downregulated upon the induction of C/EBPδ (Table 1). In order to show the association with C/EBPδ expression, genes that were confirmed to be deregulated in the same manner on qPCR as in the RNAseq data set were targeted for knockdown (KD) by short hairpin RNAs (shRNAs) in the MIA PaCa-2 cell line inducible for C/EBPδ expression (Figure S1).

To test whether these genes indeed regulate migration in PDAC cells, we subjected the established knockdown (KD)-cell lines to scratch migration assays without inducing C/EBPδ and compared the resulting migration profiles with those of the parental cell line with baseline C/EBPδ expression (without doxycycline). Comparing these data showed that the knockdown of Epidermal Growth Factor Receptor (*EGFR*) or Gelsolin (*GSN*) expression significantly reduced the migratory capacity of MIA PaCa-2 cells, implying that C/EBPδ induces suppression of these genes to temper migration (Figure 6B). To test this hypothesis further, we induced C/EBPδ in the parental cell line and in the *EGFR*-KD and *GSN*-KD daughter cell lines to test whether *EGFR*-KD or *GSN*-KD cause a more pronounced effect on the migration reduction than C/EBPδ alone. Interestingly, we did not find an additive effect of C/EBPδ induction together with *EGFR*-KD or *GSN*-KD on migration (Figure 6C). Thus, if C/EBPδ cannot further suppress *EGFR* or *GSN*—because the gene is already suppressed by an shRNA—it does not exert a further reduction in migration via other pathways. This implies that *EGFR* and *GSN* are two major downstream targets through which C/EBPδ regulates migration in MIA PaCa-2 cells.

## 4. Discussion

Lymph node and distant metastases are the main causes of cancer-related death. As at the time of diagnosis, PDAC regularly presents with metastatic disease, it is crucial to better understand the mechanisms underlying metastasis formation in this disease. We previously showed that higher tumor-cell C/EBPδ expression correlates with a lower likelihood of lymph node involvement, implying that C/EBPδ plays a tumor-suppressive role in this cancer. The aim of this study was to delineate the mechanism by which C/EBPδ reduces the formation of lymph node metastases using models of different steps of the metastatic cascade. Here, we show that C/EBPδ regulates the migratory capacity of pancreatic cancer cells, a prerequisite to initiate the metastatic cascade, by modulating the expression of genes that are essential for cell motility.

Using RNA-sequencing we found that C/EBPδ induces gene signatures associated with cell motility and locomotion. To determine whether C/EBPδ in fact affects cell motility in vitro, we used scratch wound and chemotaxis assays and found that C/EBPδ indeed significantly reduced PDAC cell migration. Importantly, when assaying the migratory capacity of a cell population, the issue of cellular heterogeneity arises. Although our cell population was derived from a small number of clones, cell-intrinsic heterogeneities cannot be entirely eradicated [40,41]. This intra-clonal heterogeneity is influenced by different factors including gene expression noise, cell cycle state, or cellular ATP-levels and leads to a heterogeneous motile and phenotypical profile of a cell population [26]. We indeed observed a large heterogeneity in the cells’ propensity to migrate, especially in MIA PaCa-2 cells. As faster moving cells are considered the most aggressive ones, we focused on the migration velocity of these cells instead of the population average and showed that C/EBPδ is a potent inhibitor of migration in highly motile cells. In PANC-1 cells, this approach did not show an effect of C/EBPδ on migration. Notably, PANC-1 cells are intrinsically not very motile in vitro despite their classification as mesenchymal subtype cells. Adding a chemotactic gradient to better resemble the conditions experienced *in vivo*, allowed us to show a migration-limiting effect of C/EBPδ also in these rather stationary cells.

In the field of cancer cell migration, different signatures that associate with cell motility and migration have been delineated. A study by Chen et al., for instance found that highly migratory breast cancer cells express high levels of EMT and Cancer Stem Cell (CSC) markers that likely account for their high migratory capacity [42]. Although we observed a phenotype suggestive of EMT, most classical EMT markers did not change in response to C/EBPδ (Figure S8); only the epithelial marker *CDH1* was strongly upregulated by C/EBPδ. (Figure S9). *CDH1* is frequently lost in PDAC cells, which associates with worse clinical features including lymph node metastases, and a poor prognosis [43]. The observation that C/EBPδ contributed to the re-expression of *CDH1* suggests that C/EBPδ not only regulates tumor cell aggressiveness through altering the actin-cytoskeletal dynamics via *GSN* and *EGFR* but also through the upregulation of cell-cell adhesion molecules such as CDH1, which limits the cell’s ability to dissociate from the primary tumor [44]. The lack of changes in mesenchymal marker expression upon activation of C/EBPδ or CDH1 suggests that C/EBPδ-induced CDH1 merely induces a partial Mesenchymal-to-Epithelial Transition (MET) in these cells, a phenomenon that has similarly been described in breast cancer [45]. Likewise, in the CSC panel, most markers remained unaltered, except Nestin (*NES*), which was non-significantly downregulated upon the induction of C/EBPδ (Figure S10). Another study by Feng et al., discusses a migration-associated signature in osteoclasts in giant-cell tumors and reveals four major signaling pathways involved with migration in these cells: *RANKL, PARs, CD137,* and *SEMA3* [46]. Subsequent testing of a panel derived from their work again showed that C/EBPδ in PDAC cells does not constitutively induce either of these pathways (Figure S11).

Although EMT and CSC-traits are two major mechanisms involved with tumor-cell migration, we found that C/EBPδ does not fully suppress either of these signatures in PDAC cells. Instead, C/EBPδ likely activates and suppresses individual targets that exert a cumulative effect on cell motility. In PDAC specifically, C/EBPδ appears to induce a partial reversal of EMT through the upregulation of *CDH1,* while mesenchymal markers remain expressed. Putatively, through *CDH1*-induction, dissemination from the primary tumor might be inhibited and migration towards distant sites is limited.

Closer investigation of some DEGs revealed that *EGFR* and *GSN* are suppressed by C/EBPδ, while the suppression of *EGFR* or *GSN* independently also significantly reduced migration. Interestingly, studies have shown that GSN is required for EGF-stimulated motility and that EGFR-mediated migration is especially sensitive to fluctuations in GSN levels, implying that both targets act within the same pathway [47,48]. GSN severs and caps actin filaments to enhance cytoskeletal remodeling [49,50]. It is overexpressed in metastatic PDAC and associates with a decreased survival [35,36]. EGFR is a main regulator of epithelial tissue development, but hyperactivity of EGFR signaling associates with enhanced tumor growth, migration, and invasion and promotes the formation of metastases [51,52,53,54]. It was previously shown that inhibiting *GSN* or EGFR through RNA interference or with antibodies and pharmacological inhibitors, respectively, reduced migration in PDAC cell lines [33,36]. Here, we confirmed these findings by targeting *GSN* and *EGFR* using shRNAs and showed a reduction in PDAC cell motility. Altogether, these findings imply that the GSN/EGFR-axis is an important pathway through which C/EBPδ regulates PDAC cell migration.

Given that C/EBPδ is known as a transcriptional activator, it may be surprising that we only identified negatively regulated genes as downstream targets to regulate migration. The observed morphological and behavioral changes induced by C/EBPδ suggest, however, that C/EBPδ promotes an epithelial cell fate. The transition from mesenchymal-like to epithelial-like states prompts further alterations in the gene expression profile. Among those might be *GSN* and *EGFR*, which would thus be indirectly regulated by C/EBPδ through the induction of an epithelial fate. An alternative regulatory mechanism might lie in the direct binding of C/EBPδ to the *GSN* and *EGFR* promoters. We found that both genes have predicted transcription factor binding sites for C/EBPα, C/EBPβ, and C/EBPγ in their promoter region but not for C/EBPδ [55]. This holds implications about the mechanism via which C/EBPδ regulates these targets genes. Although the binding site of C/EBP-proteins is highly redundant, the affinity of different family members differs in a sequence- and phosphorylation-state dependent manner [56]. C/EBPδ is likely a less-efficient transcriptional activator of *GSN* and *EGFR* than C/EBPα, C/EBPβ, C/EBPγ, or its homo- and heterodimers. A high abundance of C/EBPδ might thus compete with the more efficient binding of C/EBPα-, C/EBPβ-, and C/EBPγ-dimers and thereby hamper the proper transcriptional activation of these genes. It is unclear how high C/EBPδ must be expressed to tip over the balance with the different C/EBP-family members and, probably, this also depends on the recruitment and availability of co-transcriptional activators. The delicacy of this balance and the presence of transcriptional competitors and co-activators might further explain the contradictory effects of C/EBPδ observed in different biological contexts and cancers. However, more research is required to better understand the network of different C/EBP-family members and their interplay in health and disease.

In addition to the migration-limiting effects of C/EBPδ presented here, we previously showed that C/EBPδ also reduced proliferation and clonogenicity in PDAC cell lines [15]. This may give the impression of C/EBPδ being a universal target to control different aspects of tumorigenesis. It is indeed tempting to speculate that the retarding effects of C/EBPδ on the actin cytoskeleton not only affect cell morphology and motility but also proliferation. In fact, the actin cytoskeleton plays a vital role in different steps of cell division [57]. In adherent cells, it is required to induce the drastic morphological changes prior to division, i.e., cell detachment from a substratum and rounding of the cells. It is then involved with mitotic spindle assembly, necessary for the formation of a contractile ring for cytokinesis, for segregation of the chromosomes, and finally to restore a functional cell shape in the resulting daughter cells. In cancer cells, these processes run uncontrolled, the cells show an increase in contractility and stress fibers and the dysregulation of actin dynamics often results in multinucleation or aneuploidy caused by untimely cytokinesis [57]. Restraining the abnormal agility of the actin cytoskeleton in cancer cells thus might provide a way to reduce tumorigenicity from various angles, and C/EBPδ could be one major effector of this process.

This study is not free of limitations, one of which is background mRNA expression of the gene of interest, *CEBPD*, even in the absence of doxycycline. This effect of the Tet-On system is referred to as ‘leakiness’ and has been described before [27]. Presumably, the resulting elevated baseline expression of C/EBPδ in the inducible cells is responsible for observed baseline differences in cell shape and behavior of inducible and CTRL cells in the absence of doxycycline. Removing this leakiness would likely result in more pronounced differences such as those observed between CTRL cells in the absence of doxycycline and inducible cells in the presence of doxycycline.

Although we propose that the regulation of *GSN* and a—potentially *GSN*-dependent—*EGFR* mechanism is a key pathway through which C/EBPδ regulates migration, the rather small curated list of suggested C/EBPδ-regulated migration-associated genes could be extended further. One might consider Chromatin Immunoprecipitation (ChIP)- or Thiol (SH)-Linked Alkylation for the Metabolic sequencing of RNA (SLAM)-sequencing experiments to take a closer look at C/EBPδ’s direct transcriptional targets and to further clarify the picture of C/EBPδ’s downstream pathways.

Although we could conclude that C/EBPδ has major effects on cell morphology and motility but not on ECM-invasion or adherence and extravasation through endothelial monolayers, the above presented data do not conclusively rule out effects of C/EBPδ on other mechanisms that play a role in tumor and metastasis development. To formally exclude a role of C/EBPδ in other steps of the metastatic cascade, and to confirm the above presented effects on migration in relation to the formation of metastases, more research is needed, ideally in *in vivo* models. To this end, spontaneous metastasis models would need to be employed. Unfortunately, C/EBPδ cannot yet be re-expressed pharmacologically, whereas current xenograft models only metastasize poorly.

## 5. Conclusions

We have shown that the induction of C/EBPδ expression in PDAC cells reduces cell migration and changes cell morphology into a more epithelial-like state. We propose that C/EBPδ exerts this role through alterations of the actin cytoskeleton and suggest that *GSN* and *EGFR* are two major mediators of this mechanism. Ultimately, the reduction of migration and EMT-like phenotypes through C/EBPδ might account for the observation that PDAC patients with high primary tumor-cell C/EBPδ are less likely to develop lymph node metastases.

## Figures and Tables

**Table 1:** Selection of migration-associated genes deregulated by C/EBPδ. Log_2_ differences in gene expression and *p*-values are assessed individually between non-induced cells and C/EBPδ-inducible cells 24 h after C/EBPδ-induction using ANOVA in the Genomic Analysis and Visualization Platform R2 [20].

Gene Name	Accession No.	Log_2_ Fold Change	*p*-Value
AXL Receptor Tyrosine Kinase *(AXL)*	NM_001699	−0.62	7.5 × 10^−6^
Epidermal Growth Factor Receptor *(EGFR)*	NM_005228	−0.74	2.72 × 10^−7^
ETS Proto-Oncogene 1 *(ETS1)*	NM_005238	−0.4	5.16 × 10^−5^
Gelsolin *(GSN)*	NM_000177	−0.5	7.25 × 10^−4^
Integrin Subunit beta 4 *(ITGB4)*	NM_000213	−0.62	6.53 × 10^−6^
Myopalladin *(MYPN)*	NM_032578	−0.68	8.67 × 10^−5^
Neuroepithelial Cell Transforming 1 *(NET1)*	NM_005863	−0.51	1.41 × 10^−5^

## Data Availability

All relevant data are within the paper and its supporting information files. Sequence libraries are publicly available through the National Center for Biotechnology Information Gene Expression Omnibus under the following accession number: GSE214028.

## Supplementary Materials

The following supporting information can be downloaded at: https://www.mdpi.com/article/10.3390/cells11213334/s1, Supplementary File S1: GO term enrichment analysis; Table S1: Primers used for RT-qPCR Analysis; Table S2: Gene Sets: Positive Regulation of Actin Dynamics; Figure S1: shRNA-mediated knockdown of migration-associated C/EBPδ target genes; Figure S2: C/EBPδ does not affect PANC-1 migration in scratch wound assays; Figure S3: C/EBPδ-inducible cells show enhanced levels of *CEBPD* mRNA in the absence of doxycycline; Figure S4: C/EBPδ does not affect random single cell migration in PANC-1; Figure S5: C/EBPδ does not significantly affect the invasive capacity of PDAC cells in Matrigel; Figure S6: CAM-invasion by MIA PaCa-2 cells is unaffected by C/EBPδ; Figure S7: C/EBPδ does not alter the frequency of MIA PaCa-2 cells attaching to endothelial monolayers; Figure S8: EMT marker panel; Figure S9: *CDH1* mRNA is induced upon induction of C/EBPδ in doxycycline-inducible cells; Figure S10: CSC marker panel; Figure S11: Osteoclast migration marker panel.
