# Supplementary material for: C/EBPδ Suppresses Motility-Associated Gene Signatures and Reduces PDAC Cell Migration
Source: Cells. 2022 Oct 22;11(21):3334. doi: 10.3390/cells11213334 (PMC9655908; doi:10.3390/cells11213334)
Supplement: Supplementary file 1 [file cells-11-03334-s001.zip › cells-1964246-SM.pdf]

Supplementary Data

6. Supplementary Materials and Methods

6.1 Transwell Invasion Assay

24-well FluoroBlok transwells (#351152, Corning) were coated with Matrigel (#356234, Corning) diluted 1:2 in complete medium and allowed to solidify for 30 minutes at 37 °C. Then, 0.2×10^6 cells, inducible and CTRL cells mixed at equal ratios, were seeded on top of the Matrigel in 300 µl of plain medium with 1 % FCS and with or without doxycycline 2 µg/ml. In the bottom of the well, 500 µl complete medium with 10% FCS was added as attractant to motivate cell invasion. The bottom of transwell membranes were imaged after 42 hours using the EVOS FL Cell Imaging System at 10× magnification. Three areas per transwell were randomly selected and cells were counted using the count tool in Adobe Photoshop (version 2021 23.1.0).

6.2 Chicken Chorioallantoic Membrane (CAM) Model and Analysis of Organs

Fertilized chicken eggs were allowed to acclimatized to room temperature overnight. The next day (embryonic development day 1, EDD0), eggs were placed horizontally at 37 °C and 70 % humidity in a rotating incubator tilting by 90° twice every hour (FIEM MG140/200 Rurale) for 3 days. At EDD4, a 2 mm^z hole was created in the egg shell at the pointed end of the egg, covered with adhesive tape (Scotch Magic Tape) and eggs were placed in a vertical position. At EDD7, holes were enlarged to 1.5 cm^2 and at EDD8, orthodontic dental elastic rings (non-latex, Ø 9.5 mm) were placed on the chick chorioallantoic membrane (CAM), 2 hours prior to cell grafting. PDAC cells were trypsinized and resuspended at 40×10^6 cells/ml in Matrigel supplemented with doxycycline 2 µg/ml or not, and 50 µl of the suspension (2×10^6 cells) were grafted on each CAM. After the Matrigel had solidified, grafts were treated with either complete medium or with complete medium supplemented with doxycycline 2 µg/ml. Treatment and media were refreshed every 2-3 days. To harvest the CAMs between EDD9 and EDD13, the eggs were placed at 4 °C for 30 minutes and subsequently, the CAMs were excised using scissors, washed once in PBS and fixed in 4 % formaldehyde (#415694, Actuall Chemicals) for 1 hour at 4 °C followed by infusion with 25 % sucrose (#S0389, Merck) in PBS for 1 hour at 4 °C. CAMs were then embedded in optimal cutting temperature compound (#IA018, Tissue-Tek O.C.T. compound, ProSciTech) and sectioned using the Epredia CryoStar NX70 (#14-071-407, Thermo Scientific) while setting the specimen holder to -30 °C and the knife to -35 °C. To visualize CAMs, sections were washed in PBS, blocked with Ultra V Block (#TA-125-UB, Thermo Scientific) for 10 minutes at room temperature, and stained for Laminin (Rabbit-anti-Laminin 1:500 #NB300-144, Novus Biologicals) in BrightDiluent (#UD09-500, Immunologic) at 4 °C overnight. The next day, CAMs were washed and incubated with Alexa Fluor 594 goat anti-rabbit IgG (H+L) (#A11037/10474352, Invitrogen) for 30 minutes at room temperature. Slides were then washed again and coverslips were mounted using ProLong Gold Antifade Mountant (#P10144, Thermo Scientific). CAM integrity was visualized using the EVOS FL Cell Imaging System. To analyze lungs and liver for tumor cell invasion a grafting density of 10,000,000 cells/ml in 50 % Matrigel diluted in plain medium was used. Chick embryos were sacrificed at EDD17 by placing eggs at 4 °C for 30 minutes. Organs were harvested, snap-frozen in liquid nitrogen and homogenized in NaCl using a rotor-stator homogenizer. 100 µl of the resulting suspension were processed for RNA extraction and RT-qPCR was used to assess the presence of mCherry or mVenus mRNA using the primers listed in **Table 1**. For fluorescence activated cell sorting (FACS) analysis, one fifth of liver and lung tissue was washed in PBS, minced with a scalpel and dissociated using

an enzyme dissociation buffer (1 mg/mL Type VII collagenase (#9001-12-1, Merck), 2 mg/mL Dispase II (#42613-33-2, Merck) 1 mg/mL trypsin inhibitor (#J60982.03, Thermo Scientific), 1 unit/mL DNase I (#M0303, New England BioLabs)) at 37 °C for 1 hours. Samples of 500 µl were taken every 15 minutes and subjected to FACS analysis as described below.

6.3 Tumor Cell Extravasation and ECIS

Channels of a µ-slide flow chamber (#80606, Ibidi) were coated with 0.1% gelatin, followed by seeding a monolayer of primary human pulmonary microvascular endothelial cells (PMVEC) as described by Manz *et al. [28].* Tumor cells (C/EBPδ-inducible and CTRL in the presence or absence of doxycycline) were detached using TripLE Express Enzyme (#12604013, Gibco) and resuspended in Endothelial Cell Medium (#1001, ScienCell) at around 500,000 cells/ml. Cell suspensions were perfused over the endothelial monolayer with a flow rate of 5.8 ml/hour, corresponding to 0.5 dyn, using a syringe pump. Per condition, images of three regions of interest were acquired before and after 1 hour of perfusion using a fluorescent microscope (Lumascope 720, Etaluma) and the Lumaview software (Lumaview 720/600-series, Etaluma). Before-and-after images were exported and attached tumor cells were quantified manually.

Disruption of endothelial barrier function through pre-conditioned medium from MIA PaCa-2 cells with baseline or induced C/EBPδ was measured over 24 hours using Electric Cell-substrate Impedance Sensing (ECIS). PMVEC were seeded on a 0.1% gelatin coated 96-well plate (96W10idf PET) suitable for ECIS. Cells were maintained in endothelial cell medium (#1001, ScienCell) supplemented with 1% Penicillin/Streptomycin, 1% endothelial cell growth supplement (ECGS), 5% FBS and 1% non-essential amino acids (#X055-100, Biowest), with medium changes every other day. Electrical impedance between cell-cell interactions was measured at 4000 Hz for 24 hours. Assays were run on the ECIS Z-Theta instrument (Applied BioPhysics) as described by Szulcek *et al*. [29].

7 Supplementary Results

Table S1 – Primers used for RT-qPCR Analysis

Gene and primer orientation	Primer sequence
AXL forward primer	5′-GTGGGCAACCCAGGGAATATC-3′
AXL reverse primer	5′-GTACTGTCCCGTGTCGGAAAG-3
CEBPD forward primer	5′-GCAGAAGTTGGTGGAGCTGT-3′
CEBPD reverse primer	5′-TTACCGGCAGTCTGCTGTC-3′
EGFR forward primer	5′-AGGCACGAGTAACAAGCTCAC-3′
EGFR reverse primer	5′-ATGAGGACATAACCAGCCACC-3′
ETS1 forward primer	5′-GATAGTTGTGATCGCCTCACC-3′
ETS1 reverse primer	5′-GTCCTCTGAGTCGAAGCTGTC-3′
GSN forward primer	5′-AGATGGACTACCCCAAGCAGA-3′
GSN reverse primer	5′-GGTCCCGCCAGTTCTTGAA-3′
ITGB4 forward primer	5′-CTCCACCGAGTCAGCCTTC-3′
ITGB4 reverse primer	5′-CGGGTAGTCCTGTGTCCTGTA-3′
MYPN forward primer	5′-CTCACTGACCATTGCGGAAG-3′
MYPN reverse primer	5′-GCAGAAGTCGAATCTGTCCCA-3′
NET1 forward primer	5′-ACACCCGCCAAGAGAAGGA-3′
NET1 reverse primer	5′-TGTTCACCTCGGGACATTTCATA-3′
RPLP0 forward primer	5′-GGCACCATTGAAATCCTGAGTGATGTG-3′
RPLP0 reverse primer	5′-TTGCGGACACCCTCCAGGAAGC-3′
TBP forward primer	5′-ATCCCAAGCGGTTTGCTGC-3′
TBP reverse primer	5′-ACTGTTCTTCACTCTTGGCTC-3′
mVenus forward primer	5′-CTCGTCCATGCCGAGAGTGA-3′
mVenus revers primer	5′-CGAGAAGCGCGATCACATGG-3′
mCherry forward primer	5′-GAACGGCCACGAGTTCGAGA-3′
mCherry reverse primer	5′-CTTGGAGCCGTACATGAACTGAGG-3′

Table S2 – Gene Sets: Positive Regulation of Actin Dynamics

ABI2	*CCL21*	*EVL*	*LPAR1*	*PRKCE*	*SYNPO2*
ABL1	*CCL24*	*F2RL1*	*MAGEL2*	*PTK2B*	*SYNPO2L*
ACTN2	*CCL26*	*FCHSD1*	*MLST8*	*PXN*	*TACR1*
ALOX15	*CCL27*	*FCHSD2*	*MTOR*	*PYCARD*	*TEK*
APOA1	*CCN2*	*FER*	*MTSS1*	*RAC1*	*TENM1*
ARF6	*CCR7*	*FERMT2*	*MYOC*	*RAPGEF3*	*TESK1*
ARHGEF10	*CD47*	*FES*	*NCK1*	*RGCC*	*TGFB3*
ARHGEF10L	*CDC42*	*FHOD1*	*NCK2*	*RHOA*	*TGFBR1*
ARHGEF15	*CDC42EP1*	*FMN1*	*NCKAP1*	*RHOC*	*TPM1*
ARHGEF5	*CDC42EP2*	*GPR65*	*NCKAP1L*	*RICTOR*	*TRIM27*
ARPC2	*CDC42EP3*	*GRB2*	*NF2*	*ROCK2*	*TSC1*
BAG4	*CDC42EP4*	*GSN*	*NPHS1*	*S100A10*	*VASP*
BAIAP2	*CDC42EP5*	*HAX1*	*NRP1*	*SCIN*	*VIL1*
BAIAP2L1	*CDK5*	*HCK*	*NTF3*	*SDC4*	*WAS*
BAIAP2L2	*CFL2*	*HCLS1*	*NTRK3*	*SEMA5A*	*WASF1*
BCAS3	*CRACD*	*HRAS*	*PAK1*	*SERPINF2*	*WASF2*
BIN1	*CSF3*	*ID1*	*PDXP*	*SFRP1*	*WASF3*
BRK1	*CTTN*	*ITGB1BP1*	*PFN1*	*SMAD3*	*WASHC2C*
C15orf62	*CX3CL1*	*KIRREL1*	*PFN2*	*SNX9*	*WASL*
CARMIL1	*CYFIP1*	*LIMCH1*	*PFN3*	*SORBS3*	*WDR1*
CARMIL2	*DLG1*	*LIMK1*	*PLEK*	*STAP1*	*WHAMM*
CCDC88A	*DSTN*	*LMOD1*	*PPM1E*	*SWAP70*	*WNT11*
CCL11	*EPHA1*	*LMOD2*	*PPM1F*	*SYNPO*	*WNT4*

A gene set associated with the positive regulation of actin dynamics was comprised of the publically available complete data sets 'GOPB: Positive regulation of actin filament bundle assembly (GO:0032233), GOBP: Positive regulation of actin filament depolymerization (GO:0030836), GOBP: Positive regulation of actin cytoskeleton reorganization (GO:2000251), GOBP: Positive regulation of actin nucleation (GO:0051127) and GOBP: Positive regulation of actin filament polymerization (GO:0030838). Duplicate genes were removed such that each gene is listed only once. The resulting gene set is comprised of the genes listed in the Table S2.

Figure S1

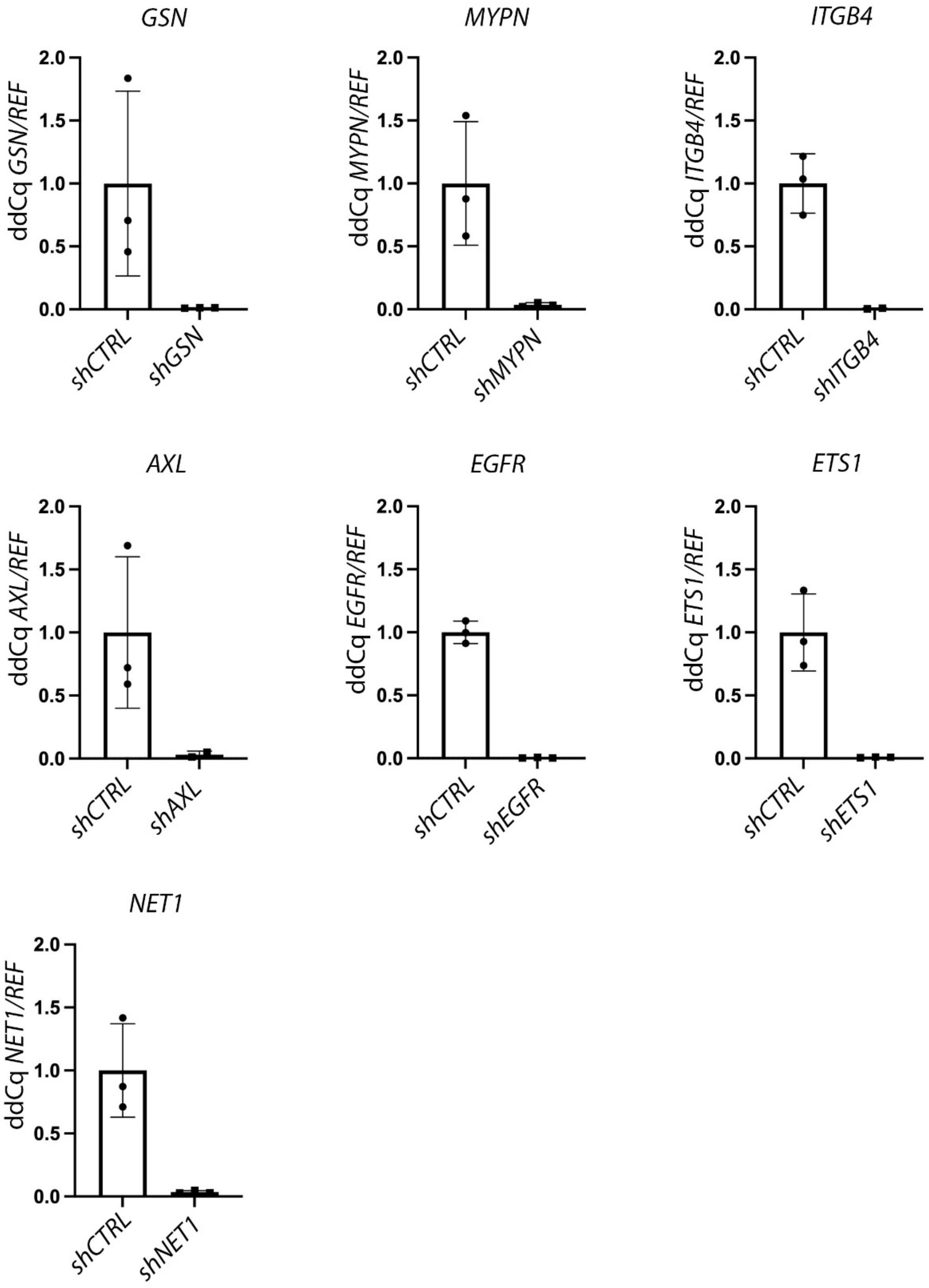

Figure S1.: shRNA-mediated knockdown of migration-associated C/EBPδ target genes is effective. *GSN, MYPN, ITGB4, AXL, EGFR, ETS1* and *NET1* were targeted with 5 separate shRNAs in the C/EBPδ -inducible MIA PaCa-2 cell line. RT-qPCR was used to select the most effective knockdown of five hairpins tested for downstream experiments. Shown is, per shRNA, the ddCq per gene, respective to *TBP* and *RPLP0*.

Figure S2

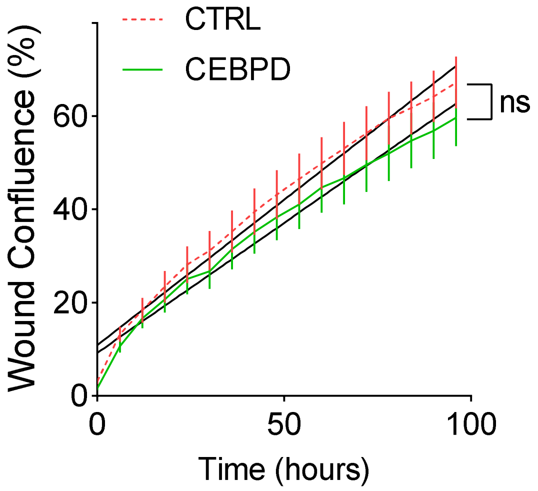

Figure S2.: C/EBPδ does not affect PANC-1 migration in scratch wound assays. PANC-1 cells were subjected to scratch wound-migration assays (four experiments with biological duplicates). Doxycycline was added to induce *C/EBPδ*. CTRL cells and *C/EBPδ* -expressing cells in the presence of doxycycline migrate at equal rates and no significant effect on migration was observed in either direction. Data were normalized to untreated cells. Straight lines were fit by simple linear regression which showed that the slopes of both curves are not significantly different (p=0.193). ns: not significant.

Figure S3

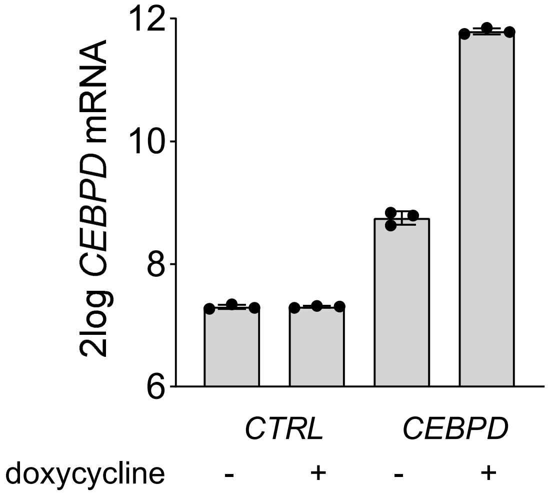

Figure S3.: C/EBPδ-inducible cells show enhanced levels of CEBPD mRNA in the absence of doxycycline. Compared to CTRL cells, cells transduced with an inducible construct for C/EBPδ expression (denoted *CEBPD*) show leakiness of the construct, i.e. enhanced *CEBPD* mRNA expression in the absence of doxycycline after 24 hours. *CEBPD* expression is more enhanced when doxycycline is added (*CEBPD* + doxycycline).

Figure S4

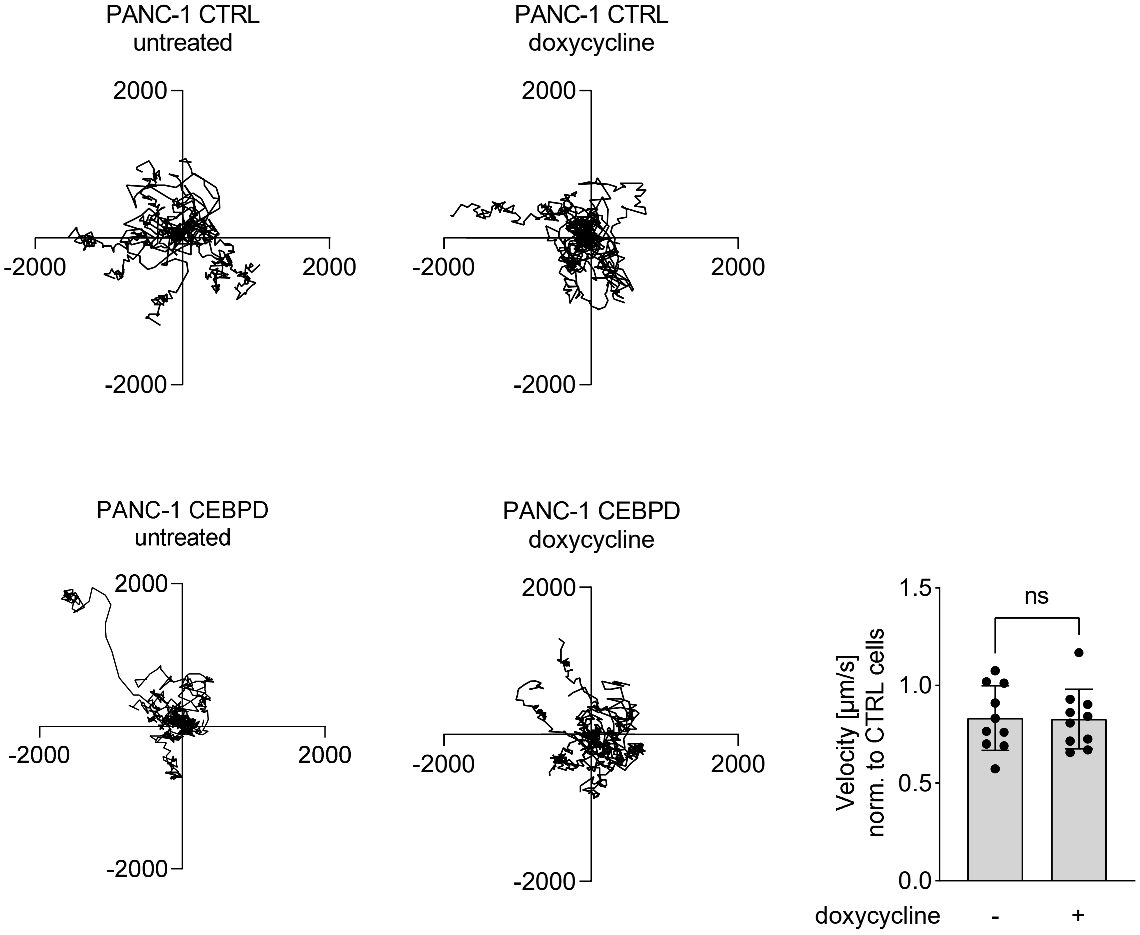

Figure S4.: C/EBPδ does not affect random single cell migration in PANC-1. Shown are single cell migration tracks and the average velocity of migration achieved by PANC-1 cells transduced with a doxycycline-inducible expression construct for C/EBPδ activation. Addition of doxycycline, i.e. induction of C/EBPδ, has no effect on random single cell migration in PANC-1.

Figure S5

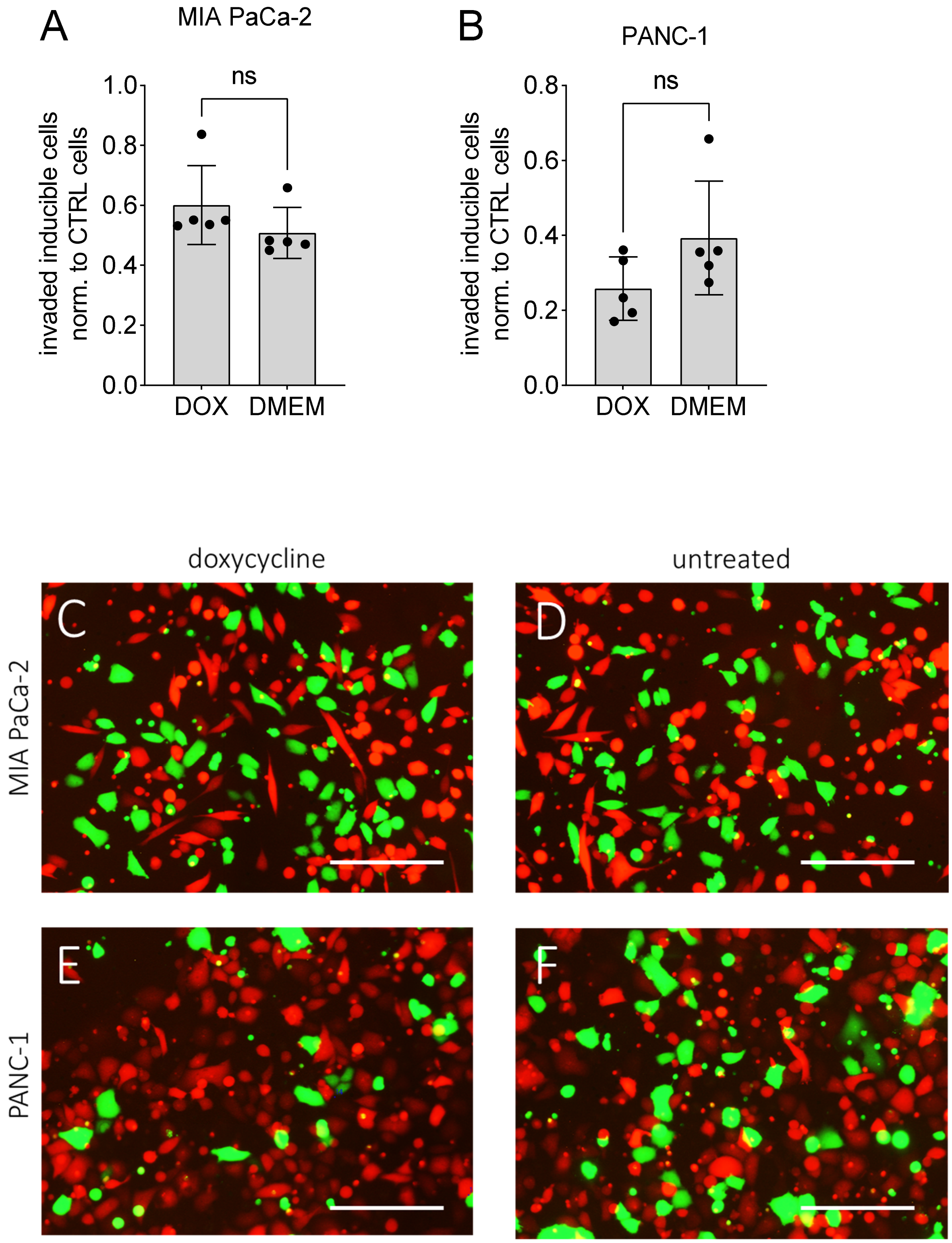
A
MIA PaCa-2
ns
invaded inducible cells norm. to CTRL cells
1.0
0.8
0.6
0.4
0.2
0.0
DOX
DMEM
B
PANC-1
ns
invaded inducible cells norm. to CTRL cells
0.8
0.6
0.4
0.2
0.0
DOX
DMEM
doxycycline
untreated
MIA PaCa-2
PANC-1
C
D
E
F

Figure S5.: C/EBPδ does not significantly affect the invasive capacity of PDAC cells in Matrigel. MIA PaCa-2 **(A)** and PANC-1 **(B)** cells inducible for CEPBD expression or not were treated or not with doxycycline and subjected to Matrigel-invasion assays. Shown is the number of inducible cells that invaded through Matrigel within 42 hours, normalized to the number of invaded CTRL cells. Induction of C/EBPδ has no effect on PDAC cell invasion through Matrigel *in vitro*. **(C-F)** show exemplary images of the invaded cells after 42 hours. Scale bars are 200 μm.

Figure S6

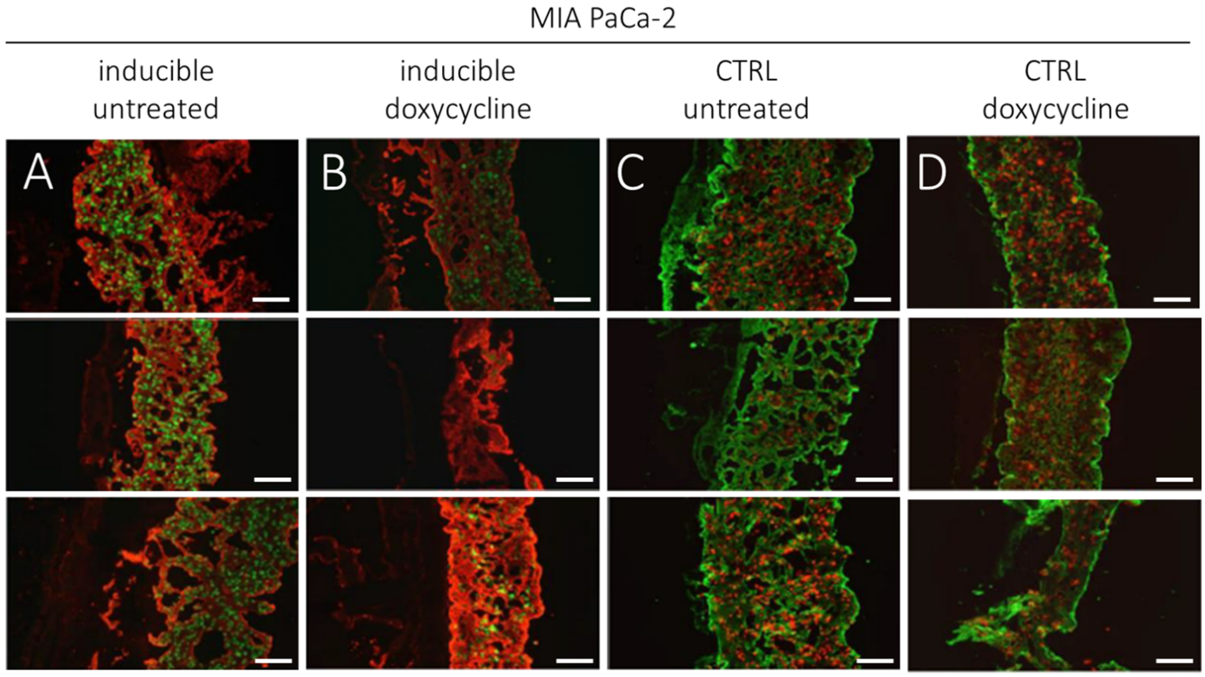

Figure S6.: CAM-invasion by MIA PaCa-2 cells is unaffected by C/EBPδ. MIA PaCa-2 cells were grafted onto CAMs in Matrigel and allowed to invade for 24 hours. Columns **A** and **B** show inducible cells (green fluorescent) invading the CAM and Matrigel (red fluorescent). Columns **C** and **D** show CTRL cells (red fluorescent) invading the CAM and Matrigel (green fluorescent). As Matrigel contains a fraction of laminin, it is also stained by the laminin-antibody. Although we observed that cells readily invade the CAM, no differences or retarding effects were observed upon the induction of C/EBPδ *in vivo*. Scale bars are 400 µm.

Figure S7

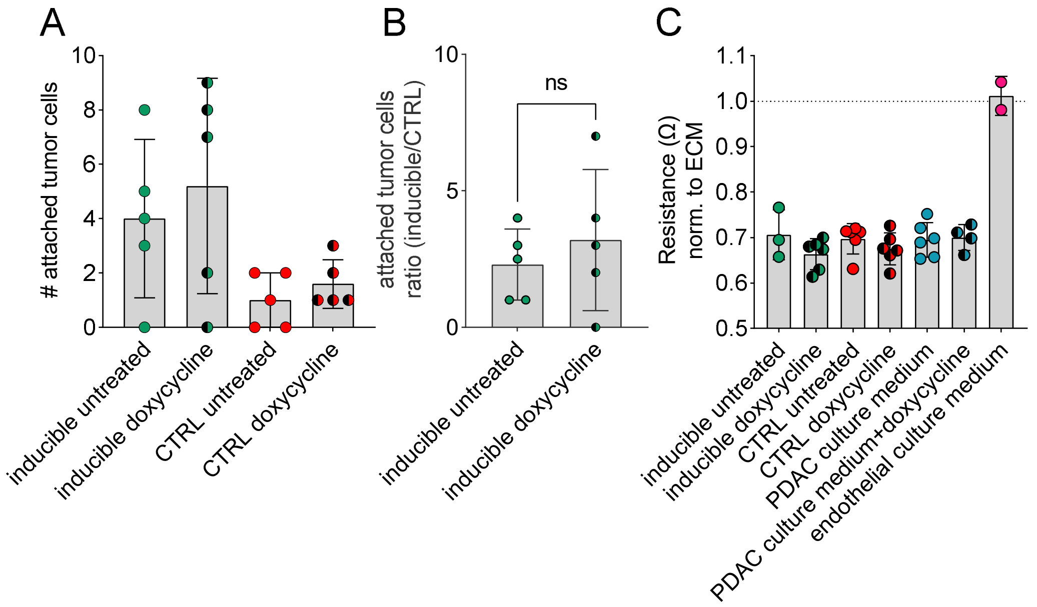

Figure S7.: C/EBPδ does not alter the frequency of MIA PaCa-2 cells attaching to endothelial monolayers. MIA PaCa-2 cells inducible for C/EBPδ expression and CTRL cells in the presence or absence of doxycycline were subjected to endothelial transfusion assays. Cell attachment was measured by manual counting of attached cells after 2 hours of transfusion. **(A)** The number of attached cells in each condition. **(B)** The ratio of inducible to CTRL cells derived from the data shown in panel (A **(C)** Electric Cell-substrate Impedance Sensing (ECIS) assays showed that medium pre-conditioned by MIA PaCa-2 cells as well as PDAC culture medium itself induce disruption of endothelial monolayers. However, this effect is unaffected by the induction of C/EBPδ. Endothelial monolayer integrity is normalized to that under endothelial cell medium (ECM) conditions.

Figure S8

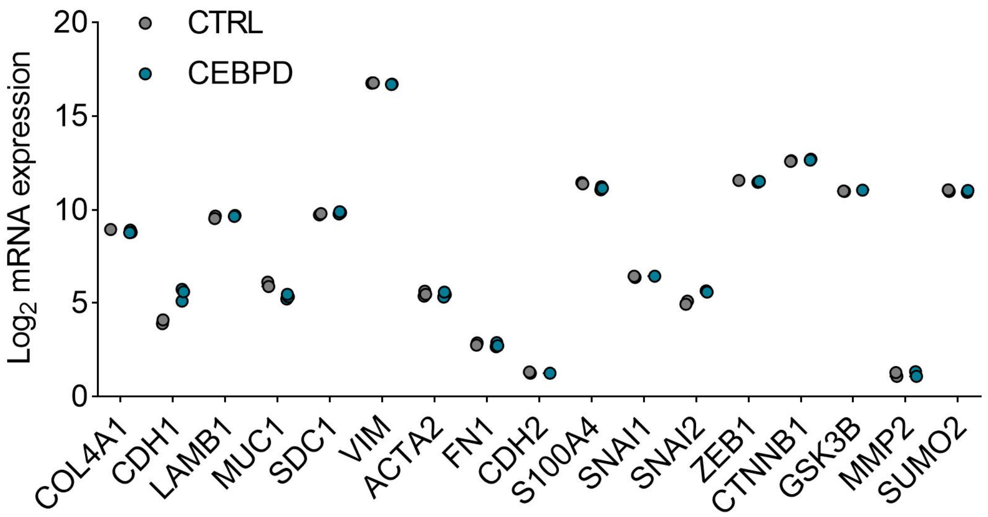

Figure S8.: mRNA expression of Epithelial-to-Mesenchymal Transition (EMT) markers are not regulated by C/EBPδ. Plotted is the Log2 mRNA expression of different markers for epithelial (*COL4A1, CDH1, LAMB1, MUC1* and *SCD1*), mesenchymal (*VIM, ACTA2, FN1, CDH2, S100A4, SNAI1* and *SNAI2*) and further major EMT players (*ZEB1, CTNNB1, GSK3B, MMP2* and *SUMO2*) in MIA PaCa-2 cells before (CTRL) and 24 hours after (CEBPD) doxycycline treatment. There is no clear tendency, i.e. up- or down-regulation of a specific group and no marker except *CDH1* changes significantly within 24 hours of C/EBPδ induction. Experiments were done using biological triplicates (N=3).

Figure S9

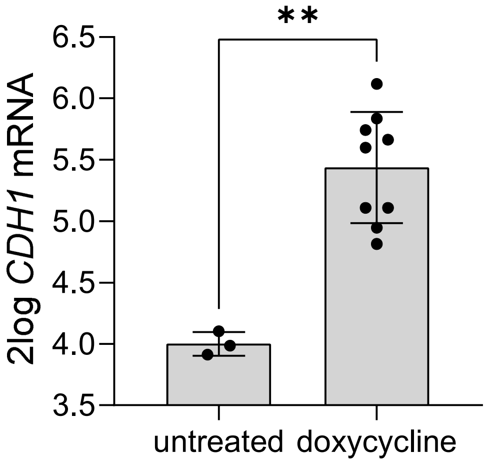

Figure S9.: CDH1 mRNA is induced upon induction of C/EBPδ in doxycycline-inducible cells. *CDH1* mRNA expression in MIA PaCa-2 cells with baseline (untreated) and induced C/EBPδ levels (doxycycline-treated for 8, 24 and 48 hours) ($p<0.01$).

Figure S10

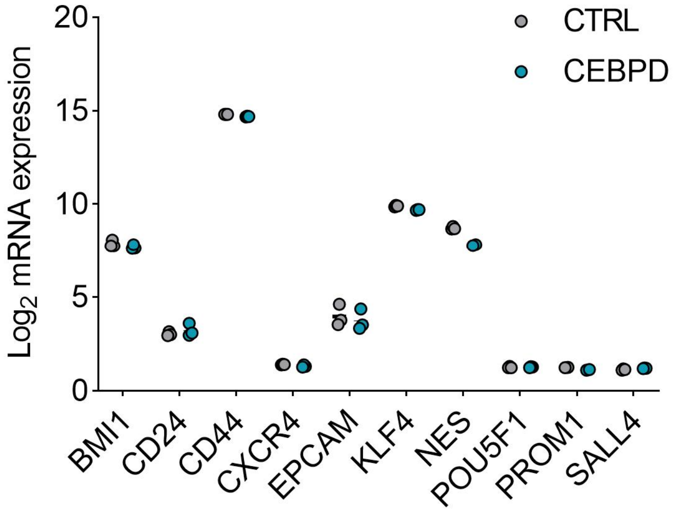

Figure S10.: mRNA expression of cancer stem cell (CSC) marker genes is not regulated by C/EBPδ. Plotted is the Log2 mRNA expression of different CSC in MIA PaCa-2 cells before (CTRL) and 24 hours after (CEBPD) doxycycline treatment. There is no clear tendency, i.e. constitutive up- or down-regulation of this panel upon C/EBPδ induction and no marker changed significantly within 24 hours of C/EBPδ induction.

Figure S11

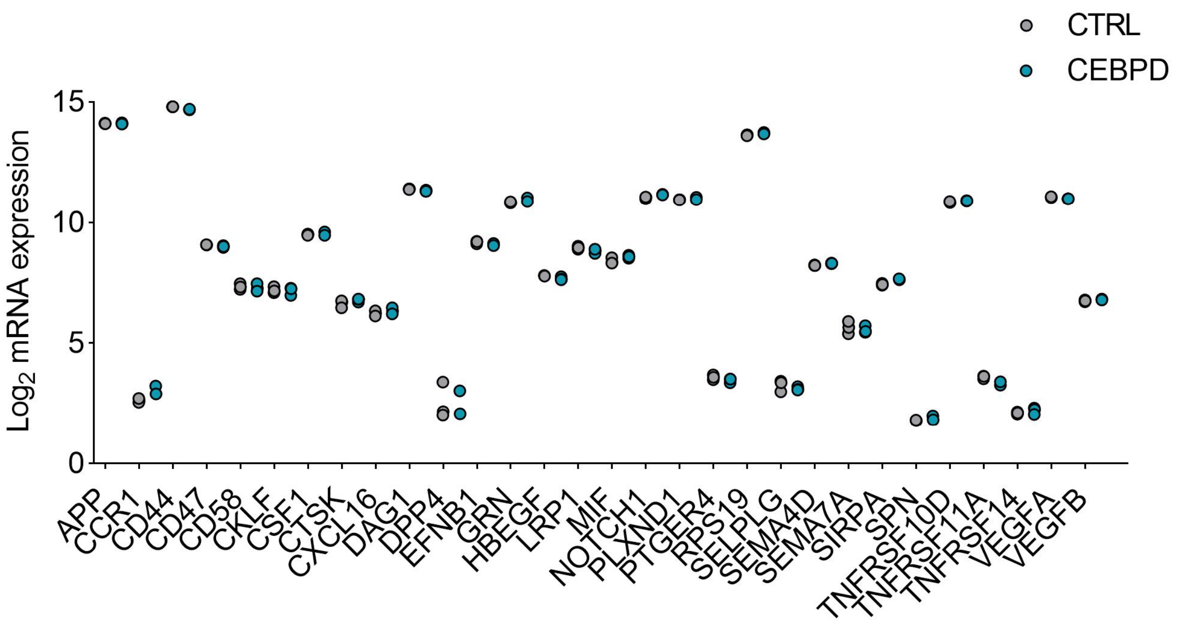

Figure S11.: mRNA expression of migration marker genes derived from osteoclast migration signature is not differentially regulated by C/EBPδ. Plotted is the Log2 mRNA expression of different markers in MIA PaCa-2 cells before (CTRL) and 24 hours after (CEBPD) doxycycline treatment. There is no clear tendency, i.e. constitutive up- or down-regulation of this panel upon C/EBPδ induction and no marker changes significantly within 24 hours of C/EBPδ induction.

References

28. Manz, X.D., et al., In Vitro Microfluidic Disease Model to Study Whole Blood-Endothelial Interactions and Blood Clot Dy-namics in Real-Time. JoVE, 2020(159): p. e61068. DOI: doi:10.3791/61068.

29. Szulcek, R., H.J. Bogaard, and G.P. van Nieuw Amerongen, Electric cell-substrate impedance sensing for the quantification of endothelial proliferation, barrier function, and motility. J Vis Exp, 2014(85). DOI: 10.3791/51300.
